# Resetting the circadian clock of Alzheimer’s mice *via* GLP-1 injection combined with time-restricted feeding

**DOI:** 10.3389/fphys.2022.911437

**Published:** 2022-08-24

**Authors:** Yanqiong Dong, Le Cheng, Yingying Zhao

**Affiliations:** ^1^ Department of Basic Medicine Sciences, School of Basic Medical Sciences, Dali University, Dali, Yunnan, China; ^2^ Department of Physiology, School of Basic Medical Sciences, Shenzhen University Health Sciences Center, Shenzhen, Guangdong, China; ^3^ BGI-Yunnan, BGI-Shenzhen, Kunming, Yunnan, China

**Keywords:** amyloid-*β*, circadian rhythm, glucagon-like peptide-1, time-restricted feeding, Alzheimer’s disease

## Abstract

Circadian rhythm disturbances are the most common symptoms during the early onset of AD. Circadian rhythm disorders aggravate the deposition of amyloid plaques in the brains of AD patients. Therefore, improving the circadian rhythm of AD patients might slow down the pathological development of neurodegeneration. Circadian regulation is driven by a master clock in suprachiasmatic nuclei (SCN) and peripheral clock located in peripheral organs. The rhythmic feeding–fasting cycle has been proved to dominant cue to entrain peripheral clocks. We hypothesized that dietary intervention to a certain period of time during the dark phase might entrain the clock and reset the disrupted daily rhythms of AD mice. In this study, exogenous glucagon-like peptide-1 (GLP-1) treatment, time-restricted feeding (TRF), and the combination were used to examine the effect of overall circadian rhythm and neurodegenerative pathogenesis of transgenic AD mice. It was confirmed that GLP-1 administration together with time-restricted feeding improves circadian rhythm of 5 × FAD mice including the physiological rhythm of the activity–rest cycle, feeding–fasting cycle, core body temperature, and hormone secretion. Furthermore, GLP-1 and TRF treatments improved the diurnal metabolic homeostasis, spatial cognition, and learning of 5 × FAD mice. The aberrant expression of clock genes, including *Baml1*, *Clock,* and *Dbp*, was improved in the hypothalamus, and pathological changes in neurodegeneration and neuroinflammation were also observed in AD mice with dual treatment.

## Introduction

Alzheimer’s disease (AD) is an age-related and irreversible metabolic neurodegenerative disease (Zhou et al., 2020). AD is characterized by significant, persistent, and progressive memory loss, usually accompanied by cognitive impairments and personality changes (Park et al., 2019; Kim et al., 2020). The major morphologically observed lesions of AD include the accumulation of amyloid plaques formed of amyloid-β (Aβ) protein and neurofibrillary tangles (NFTs) of hyperphosphorylated Tau protein (Ittner and Götz, 2011; Litvinchuk et al., 2018; Mattsson-Carlgren et al., 2020). Approximately 25–60% of AD patients exhibit diurnal rhythm disturbances and sleep–wake disorders (Reisberg et al., 1987; Musiek et al., 2018; Van Erum et al., 2018). The circadian dysrhythmia has often emerged as sun-downing syndromes (Prinz et al., 1990; Van Erum et al., 2018). Accumulating evidence indicates that disturbed sleep and circadian dysregulation have a bidirectional relationship with AD (Musiek and Holtzman, 2016; Oh et al., 2019). In addition, the disturbance of the biological clock most likely exacerbates the severity of other symptoms associated with AD, such as memory loss (Gerstner and Yin, 2010). Therefore, circadian rhythm restoration and sleep improvement are considered to be one of the promising therapeutic strategies for AD.

Circadian rhythms are endogenous physiologic cycles of approximately 24 h (Sinturel et al., 2017). Molecular clocks are operated by a series of clock genes constituting an interlocked transcriptional/translational feedback loop. *Bmal1* and Clock (or Npas2) form a heterodimer to activate Per and Cry genes *via* binding to E-box elements in their promoter regions. PER and CRY proteins accumulate in the cytoplasm and form dimers to enter the nucleus and then inhibit the transcriptional activity of the *Bmal1/Clock* (or *Npas2*) complex (Yang et al., 2013; Yang et al., 2017). *Bmal1/Clock* also activates the expression of nuclear hormone receptors *ROR* and *Rev-erb*, which regulate *Bmal1* expression (Oshima et al., 2019). Core body temperature rhythms, serum melatonin, and cortisol are considered as circadian biomarkers (Raleigh et al., 2017; Sinturel et al., 2017; Sertaridou et al., 2018). The breakdown of daily circadian rhythms has been linked to a wide range of human morbidities such as metabolic syndrome, vascular disease, and neurodegenerative diseases (Bass and Takahashi, 2010; Krishnaiah et al., 2017; Oh et al., 2018).

Circadian rhythms are generated by a central pacemaker in the suprachiasmatic nuclei (SCN) of the hypothalamus that receives environmental cues, synchronizes peripheral clocks present in the extra-SCN tissues, and regulate daily physiology and behavior (Zhao et al., 2016; Castelo-Szekely et al., 2017; Allada and Bass, 2021). It has been found that the circadian clock is entrained by light, temperature, and eating–fasting cycles (Simoni et al., 2014; Greco and Sassone-Corsi, 2019; Lewis et al., 2020).

The feeding–fasting cycle send signals to the SCN through nutrients and hormones, such as glucagon-like peptide-1 (GLP-1) and leptin (Brubaker and Gil-Lozano, 2016; Chauhan et al., 2017). Time-restricted feeding (TRF) had been suggested to a promising nonpharmacological intervention for circadian dysrhythmia. GLP-1 is produced from the “L” cell in the gastrointestinal tract (Abdulreda et al., 2016) and mediated intake-suppressive effects (Hayes et al., 2011; Zhao et al., 2012).

Various studies have revealed the hierarchical architecture of timing system. However, the effect of feeding rhythms on the central clock and peripheral clocks are still poorly understood. In our study, we hypothesized that circadian arrhythmia of AD mice might be alleviated *via* GLP-1 administration and/or time-restricted feeding. All experiments were performed with 5 × FAD mice (20 weeks) and their wildtype littermates. Our finding has demonstrated that combined TRF and GLP-1 treatment partly rescue the fragmented activity–rest and feeding–fasting cycle compared with two groups of sole medications. Furthermore, GLP-1 and TRF treatments improved the diurnal metabolic homeostasis, spatial cognition, and learning of 5 × FAD mice. Abnormal expression of clock genes, including *Baml1*, *Clock,* and *Dbp*, was improved in the hypothalamus, and ameliorated pathogenesis of neurodegeneration was also observed in AD mice with dual treatment. The study provided a novel possible treatment of AD patients with circadian rhythm disorders.

## Materials and methods

### Overall study scheme and protocol

**FIGURE 1:**
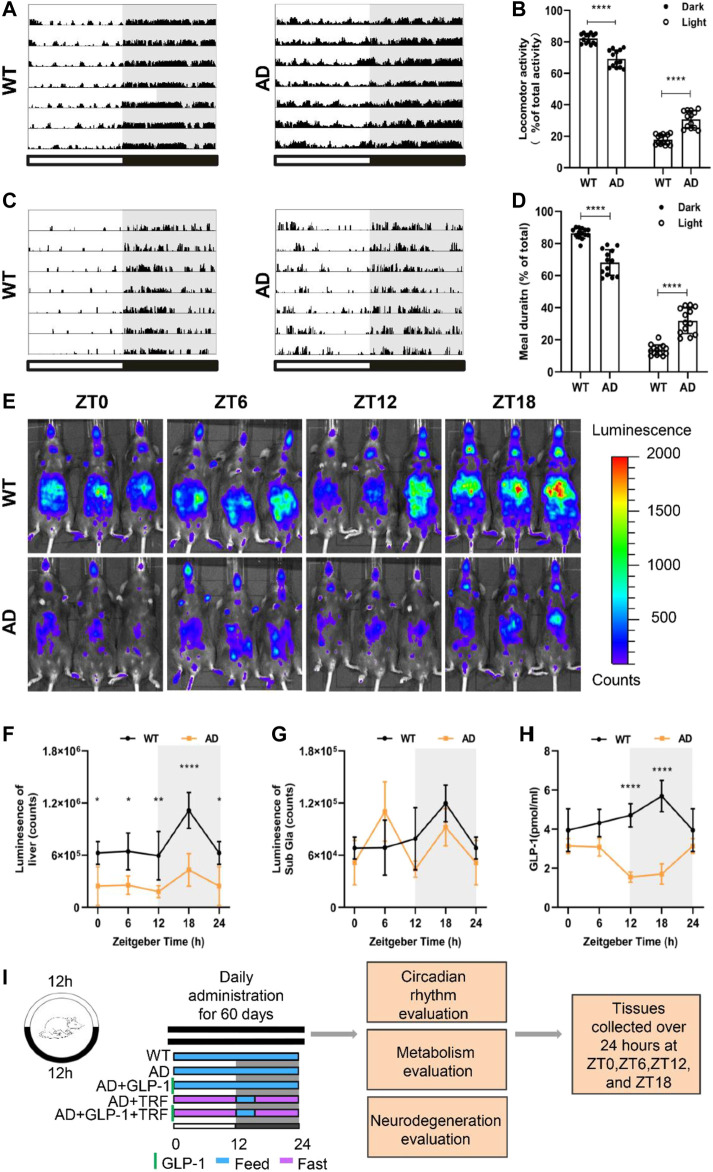
Alzheimer’s disease mice exhibit circadian rhythm disturbances. **(A)** Representative locomotor activity records of 5 × FAD mice (AD) and wild-type mice (WT), respectively. Locomotor activity was defined as the moving distance per unit time (2 min). Each horizontal line represents 24 h. Periods of darkness are indicated by grey backgrounds. The black and white bars on the button indicate 12 h-dark and 12 h-light periods, respectively. **(B)** Percentage of the locomotor activity in the dark and light/total activity (24 h). *n* = 7 per group, ****p < 0.0001, using two-way ANOVA followed by Sidak *t* test. **(C)** Representative meal duration records of two groups. **(D)** Percentage of the meal duration in the dark and light/total meal duration (24 h). *n* = 7 per group, ****p < 0.0001, using Two-Way ANOVA followed by Sidak *t* test. **(E)** Representative photographs of PER2:LUC mice *in vivo* monitoring from each time point at 6 h intervals. **(F,G)** Raw photon count data of individual bioluminescence rhythms from E. *n* = 3 per group, *n* = 3 per group, *p < 0.015, **p < 0.01 using Two-Way ANOVA test. **(H)** GLP-1 secretion levels in both groups. **(I)** The protocol design of our study.

The flow chart of the experimental design and analysis for this study is presented in Figure 1. In order to conduct this experiment, we defined 7:00 a.m. as zeitgeber time zero (ZT0) and 19:00 as zeitgeber time twelve (ZT12). Briefly, all animals are administration for 2 months. 5 × FAD mice were divided randomly into four groups (*n* = 12 per group): a control group (AD), a glucagon-like peptide-1 group (AD + GLP-1), a time-restricted feeding group (AD + TRF), and a combined treatment group (AD + GLP-1 + TRF), at the same time, with their nontransgenic wild-type (WT) littermates as control. GLP-1 (liraglutide injection, Victoza) was diluted with normal saline and injected intraperitoneally (200 ug/kg body weight, once daily) at ZT0 and TRF means that animals are given food from ZT12∼ZT16. The joint treatment represents the superposition of the two schemes. The locomotor activity is recorded. Exogenous GLP-1 was not administered to mice on the day of tissue collection.

### Animals

5 × FAD mice and PER2:LUC homozygous mice were obtained from the Jackson Laboratory. Mice were housed under hygienic conditions with a 12-h light/12-h dark cycle (temperature22∼25°C; humidity∼40%) and were provided rat–mouse standard diet in the form of pellets (GB14924.3-2010). Mice in all the groups had *ad libitum* access to water under an LD cycle during the entire study.

### Monitoring of locomotor activity and meal duration

Mice were individually housed within PhenoTyper 3,000 cages and their locomotor activity were monitored and analyzed by the EthoVision XT V14 tracking software (Noulds Information Technology). Spontaneous locomotor activity was defined as the moving distance per unit time (2 min). The mice were video-tracked for a week at least. Before starting the experiment, the mice were acclimated to the LD cycle for 2 weeks to eliminate the effects of environmental changes on the experimental results. Cosine curves were fitted to repeated measures activity and meal data using the cosinor method, and the characteristics of the curves were then calculated (Molcan, 2019).

### Monitoring of core body temperature

Core body temperature was measured using a rectal probe thermometer. The core body temperature was measured using a rectal thermometer every 3 h, with three replicates per mouse.

### *In vivo* monitoring of peripheral PER2:LUC rhythms

PER2:LUC homozygous mice carrying the PER2-fused luciferase reporter gene were crossed with 5 × FAD homozygous mice. The rhythmic expression of PER2 in their offspring mice and PER2:LUC homozygous mice was detected by *In Vivo* Imaging System (IVIS) Spectrum instrument (Perkin-Elmer, Waltham, MA, United States). The liver and submandibular gland (indicated as ‘‘Sub Gla’’ in figures) were assessed after the subcutaneous injection of D-luciferin potassium salt (luc001, Sciencelight, Shanghai, China) at a dose of 15 mg/kg body weight into PER2:LUC mice. Mice were placed on their backs for monitoring of the liver and submandibular gland. Animals were imaged approximately 8 min after the injection of D-luciferin potassium salt. The total fluorescent counts were evaluated for each group using elliptical regions of interest in Living Image Software (Perkin Elmer, Waltham, MA, United States).

### ELISA

The mouse GLP-1 ELISA kit (JL11122), mouse melatonin ELISA kit (JL10087), mouse cortisol ELISA kit (JL12086), and mouse Orexin AELISA kit (JL48032) were purchased from Jianglai Biological Industries (Shanghai, China) and used according to the manufacturer’s instructions.

### Glucose tolerance tests

Intraperitoneal glucose tolerance tests (IPGTTs) were performed 8 weeks after treatment. The mice were fasted 16 h earlier and weighed. Glucose (resuspended in PBS, filtered) was injected intraperitoneally at a concentration of 2 g/kg body weight. Blood glucose was measured with a glucometer at 0, 15, 30, 60, 90, and 120 min. After the test, the mice were given food (including time-restricted mice).

### Morris water maze tests

The MWM tests were performed starting from ZT0 according to a previously reported protocol. Briefly, a circular pool of 120 cm was filled with 22-23°C water, and a nontoxic paint was added to make an opaque and white background. A circular platform with a diameter of 10 cm was placed 1 cm below the water level. The maze was divided into four quadrants (northwest, northeast, southwest, and southeast). During the test, the pool was curtained with spatial cues arranged at fixed positions. In the acquisition phase, the mice were allowed to swim and search freely for 60 s. The mice that voluntarily found the platform were permitted to remain on the platform for 10 s, while the mice who failed to find the platform were gently guided to it and retained there for 10 s. Each mouse was released from different quadrants in each trial, and each mouse underwent four trials per day for five consecutive days. The platform was removed on day 6, and each mouse was released from the quadrant opposite to previous location of the platform and given 60 s to search in the water. After each test, the mice were gently dried with a towel. Swimming speeds, escape latencies, swimming tracks, and times crossing the target spot were recorded and analyzed using the Morris maze analysis system (ZS Dichuang, Beijing, China). The test performers and data analyzers were blinded to the mice groups.

### Novel object recognition tests

Novel object recognition tests were performed following the protocol described in the literature. Recognition memory was assessed by performing novel object recognition (NOR) tests following protocols described in the literature. One week prior to testing, the mice were handled 1–2 times a day for at least 1 minute each time. On the first day, the mice were allowed 5 min to freely explore an empty square chamber (around 40 cm × 40 cm × 40 cm) for habituation. The next day (training session), two identical objects were placed in the phase confines of the chamber at an equal distance and allowed the mice to freely explore the room for 10 min. After 24 h (test session), one object used during training session and a new object in opposite quadrants was placed. The mouse was placed in the center of the arena and allowed free exploration for 10 min. The time spent exploring familiar and novel objects was recorded for each mouse. The exploration of an object was defined as active tentacle sweeping or sniffing when the animal’s nose was within 2–3 cm of the object. The device was cleaned thoroughly with 75% ethanol after each test. A behavioral test was conducted from 8 a.m. to 4 p.m. The exploratory behavior of the mice was recorded and analyzed blindly using an automated video tracking system (EthoVision XT V14).

### Tissue sample collection and real-time qPCR

The animals were euthanized by cervical dislocation, and the organs were rapidly harvested and weighed (*n* = 3 per sampling time point every 6-h). The samples were immediately stored in dry ice. The total RNA was extracted using the Trizol method and then reverse transcribed into cDNA. The SYBR Green kit was used to determine the relative gene expression on a Biometra QPCR System. All results were normalized to that of GAPDH at ZT0. The relative gene expression was determined using the 2−ΔΔCT comparative method. The corresponding primers were synthesized by Sangon Biotech (China) as shown in Table 1.

**TABLE 1:** Primer sequences used for quantitative RT-PCR analysis.

Gene		Primer sequence
GAPDH	Forward	5′-CAT​GGC​CTT​CCG​TGT​TCC​TA-3′
	Reverse	5′-CCT​GCT​TCA​CCA​CCT​TCT​TGA-3′
Bmal1	Forward	5′-CCT​AAT​TCT​CAG​GGC​AGC​AGA​T-3′
	Reverse	5′- TCC​AGT​CTT​GGC​ATC​AAT​GAG​T-3′
Clock	Forward	5′- TTG​CTC​CAC​GGG​AAT​CCT​T-3′
	Reverse	5′- GGA​GGG​AAA​GTG​CTC​TGT​TGT​AG-3′
Per2	Forward	5′- GCG​GAT​GCT​CGT​GGA​ATC​TT-3′
	Reverse	5′- GCT​CCT​TCA​GGG​TCC​TTA​TC-3′
Per3	Forward	5′- ATG​ACA​TAC​CAG​GTG​CCC​GA-3′
	Reverse	5′-TGC​TGC​TGT​TCC​ATG​CTC​TG-3′
Dbp	Forward	5′- CGT​GGA​GGT​GCT​AAT​GAC​CTT​T-3′
	Reverse	5′- CAT​GGC​CTG​GAA​TGC​TTG​A-3′
Rev-erbβ	Forward	5′- TAC​ATT​GGC​TCT​AGT​GGC​TCC-3′
	Reverse	5′- CAG​TAG​GTG​ATG​GTG​GGA​AGT​A-3′
RORα	Forward	5′- CTT​CTT​CCC​CTA​CTG​TTC​CTT​C-3′
	Reverse	5′- TCT​CTG​CTT​GTT​CTG​GTA​GTT​T-3′

### Immunohistochemistry

3 μm-thick paraffin coronal sections of the mouse brain tissue were used for immunohistochemical analysis. Immunohistochemistry was performed according to the manufacturer’s instructions of the PV-6000D kit (Zhongshanjinqiao, Beijing, China). In detail, paraffin sections were baked at 65°C for 120 min, deparaffinized in xylene, and rehydrated through graded alcohol. Antigen retrieval was performed by microwave heating at 99°C in citrate buffer for 5 min three times and endogenous peroxidase was blunted by incubating samples with 3% H2O2 for 10 min. The sections were then placed in blocking buffer (10% natural goat serum) for 30 min at 37°C. The sections were incubated overnight at 4°C with the following primary antibodies: beta Amyloid antibody (MOAB-2) (1:1,000, NBP2-13075, Novus Biologicals), GFAP (E4L7M) XP^®^ Rabbit mAb (1:200, #80788, Cell Signaling Technology), and Iba1/AIF-1 (E4O4W) XP^®^ Rabbit mAb (1:2000, #17198, Cell Signaling Technology). After that, secondary antibody working solution was incubated at 37°C for 1 h. Finally, the slides were then dehydrated in ethanol followed by xylene and sealed with cover slips. The percent area of positive labeling was analyzed at ×20 magnification.

### Statistical analyses

All the results were expressed as the mean ± SD. One-way ANOVA and Dunnett’s multiple comparison test were conducted to determine the significance of the differences between the AD group and the other groups, while a Sidak test was used for analyzing the differences between the two groups. Two-way ANOVA analysis was applied to when there were two factors. The differences were considered significant when *p* < 0.05. The calculations were performed using GraphPad Prism 8.0.2 (GraphPad Software, San Diego, CA, United States).

## Results

### Alzheimer’s disease mice exhibit circadian rhythm disturbances

Spontaneous motor activity of WT and 5 × FAD mice was evaluated under standard light and dark conditions (LD 12:12). Consistent to our previous result (Yao et al., 2020), 5 × FAD mice exhibit pronounced circadian disturbance with altered diurnal activity–rest cycle and significant fragmented sleeping (Figure 1A). 5 × FAD mice showed a significant increase in activity during the day and a decrease at night (Dark: *p* < 0.0001, AD vs. WT; Light: *p* < 0.001, AD vs. WT. Figure 1B). Importantly, we next assessed the feeding–fasting cycle of AD mice and observed that prominent disturbed diurnal cycle with much more meal numbers and duration of AD mice (Figure 1C). Similarly, the percentage of AD mice that food intake during the day and night were different from WT and a decrease at night (Dark: *p* < 0.0001, AD vs. WT; Light: *p* < 0.001, AD vs. WT. Figure 1D). Circadian rhythms of clock gene expression in the peripheral organs of mice were noninvasively measured by fluorescence imaging of PER2:LUC and 5 × FAD hybrid to PER2:LUC mice. The bioluminescence values of the liver and submandibular gland, at 8 min after the mice were injected with fluorescence, were measured and analyzed (Figure 1E). The rhythmic signal of PER2 protein from the 5 × FAD liver decreased rhythmicity with the overall lower level and smaller amplitude (Figure 1F). The accumulation of Per2 signal from the submandibular gland during the dark phase is no longer observed for 5 × FAD mice (Figure 1G), suggesting an arrhythmic function of submandibular gland. We therefore measured the rhythmicity of peripheral hormone secretion of GLP-1. The control wildtype mice showed their highest levels of GLP-1 at ZT18, with a progressive decline to the low levels observed in the light phase. Whereas the overall basal level of GLP-1 of AD mice was deceased and exhibited altered circadian phase (Figure 1H). Interestingly, this was likely the mirror for the increased meal numbers and duration during the subjective day time. We hypothesized that time-restricted feeding (TRF) and GLP-1 administration could have alleviated circadian arrhythmia of AD. In line with this hypothesis, the master clock will be entrained *via* peripheral adjustments. The experiments were carried out with five groups animals: WT, AD (5 × FAD), AD mice with GLP-1 administration sole, AD mice with TRF, AD mice with GLP-1 administration, and TRF combined treatment (Figure 1I).

### GLP-1 and TRF treatments improved the abnormal diurnal activity/rest cycle, feeding/fasting cycle, and body temperature rhythmicity of 5 × FAD mice

**FIGURE 2:**
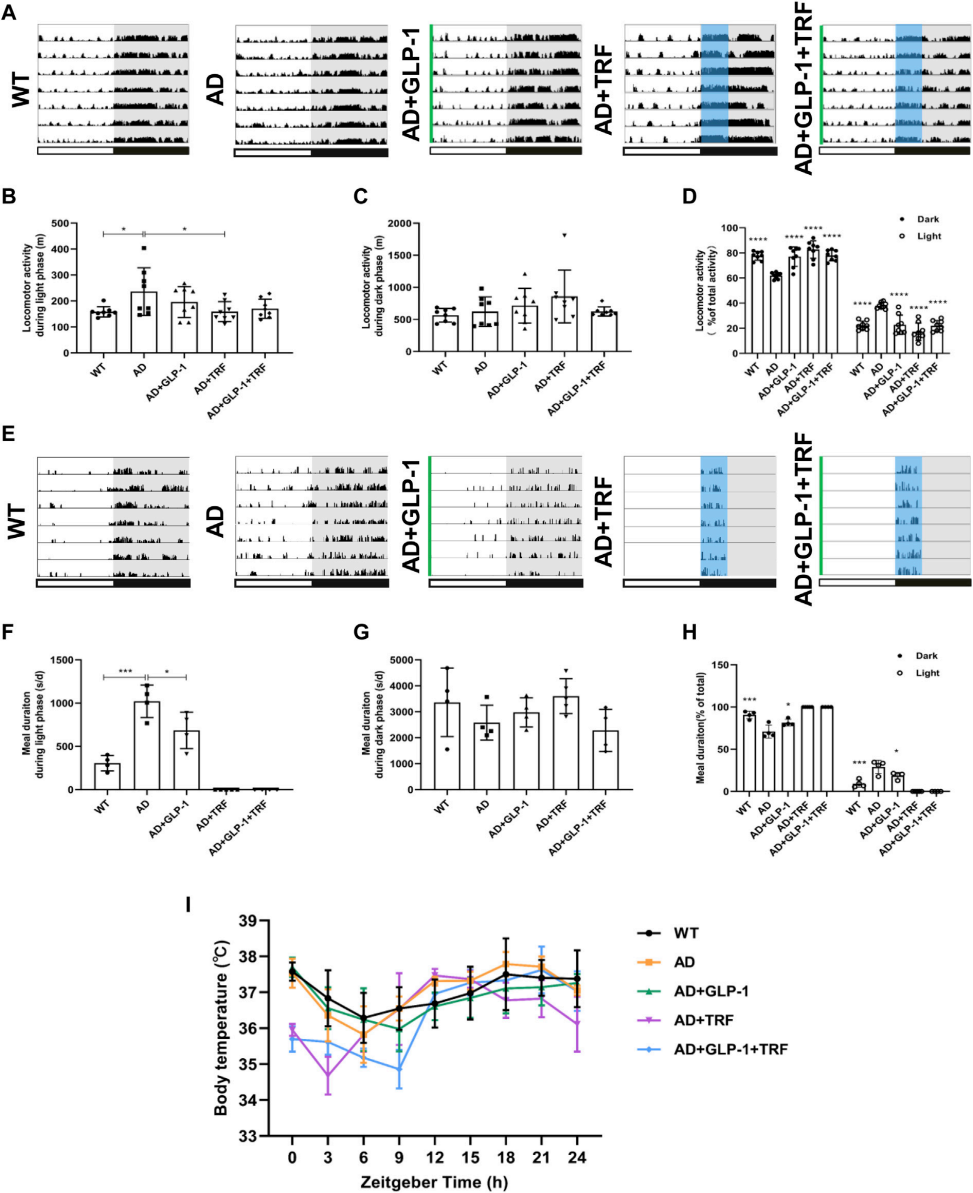
Effect of GLP-1 and TRF on circadian rhythm disorder in mice models of Alzheimer’s disease. **(A)** Representative locomotor activity records of each group. The green line represents the time of intraperitoneal injection of GLP-1 to this group of mice. The blue area delineates 4-h of feeding time. **(B)** Activity during the light phase (m). *n* = 7 per group, *p < 0.05 vs. AD group using One-Way ANOVA followed by Dunnett’s test. **(C)** Activity during the dark phase (m). **(D)** Ratio of the activity in the dark and light/total activity in each group. *n* = 7 per group, ****p < 0.0001 vs. AD mice using two-way ANOVA followed by Dunnett’s test. **(E)** Representative meal duration records of each group. **(F)** Meal duration during the light phase (s). *n* ≥ 4 per group, *p < 0.05, ***p < 0.0001 vs. AD group using One-Way ANOVA followed by Dunnett’s test. **(G)** Meal duration during the dark phase (s). *n* ≥ 4 per group. **(H)** Ratio of meal duration in the dark and light/total meal duration (24 h) in each group. *n* ≥ 4 per group, *p < 0.05, ***p < 0.0001 vs. AD mice using Two-Way ANOVA followed by Dunnett’s test. **(I)** Body temperature was measured every 3 h for 24 h continuously.

We assessed the circadian rhythms by examining and quantifying spontaneous activity in groups of mice under a 12-h light and 12-h dark conditions (LD12:12). The wildtype mice exhibited robust diurnal rhythm with a high percentage activity consolidated to the night time. Consistent with the expected results, an increased activity was associated with increased food intake (Figures 2A,E). In contrast, activity/rest cycles in AD mice suffered from disrupted patterns with fragmented rest and activity (Figure 2A), and the amount of activity changed during the day and night (Figures 2B–D). In the daytime, the AD mice appear more active. AD mice with 60 days GLP-1 administration showed distinguished profile with reduced activity during the light phase. Moreover, mice with 60 days TRF sole treatment or combination with GLP-1 also exhibited improved diurnal rhythmicity (*p* = 0.0267, AD vs. WT; *p* = 0.0280, AD vs. AD + TRF. Figure 2B). Meanwhile, the AD mice had a much higher proportion of activity during in the light, and the treated mice also had significantly altered percentages of activity during the day and night (Dark: *p* < 0.0001, AD vs. WT, AD vs. AD + GLP-1, AD vs. AD + TRF and AD vs. AD + GLP-1+TRF. Light: *p* < 0.0001, AD vs. WT, AD vs. GLP-1, AD vs. AD + TRF and AD vs. AD + GLP-1+TRF. Figure 2D). In addition, the feeding/fasting cycle was analyzed *via* recording meal duration (Figures 2E–H). In contrast, GLP-1 treatment did suppress food intake substantially during the light phase (Figures 2E,F) and improved the diurnal feed/fast rhythmicity of AD mice (Dark: *P* < 0.0002, AD vs. WT, *P* < 0.0323, AD vs. AD + GLP-1. Light: *P* < 0.0002, AD vs. WT, *P* < 0.0323, AD vs. AD + GLP-1. Figure 2H), whereas feeding/fasting cycle of the other two group AD mice was forced to eat during the subjective night with TRF treatment. At the same time, there was no statistical difference in meal duration between groups during the night (Figure 2G). Notably, the activity and feeding rhythms recorded after the injection of saline or a combination of saline and TRF in AD mice were not significantly different from AD and AD + TRF groups (Supplementary Figure S1).

Cosinor analysis was performed on the measured activity and meal data, including mesor, amplitude, acrophase, and bathyphase. The fitted cosinor curve of the activity is shown in Supplementary Figures S2A–E. In detail, the mesor of the five groups are not statistically different (Supplementary Figure S2F). The amplitude of the AD group was significantly lower than that of the WT group, slightly increased in the GLP1 treatment group, and significantly increased in the TRF combined treatment group (Supplementary Figure S2G). The acrophase and bathyphase of the AD group were higher than those of the WT group, and all the three treatment groups were significantly lower than the AD and WT groups (Supplementary Figures S2H–I). Due to the artificial restriction of the feeding time of the TRF group and the combination treatment group, we performed the feeding cycle analysis of the three groups (Supplementary Figure S3A–C). The AD group had higher mesor, and it was significantly different from the WT group and the GLP-1 treatment group (Supplementary Figure S3D). However, the amplitude, acrophase, and bathyphase of the three groups were not statistically different (Supplementary Figure S3E–G). In addition, we recorded the daily food intake of mice in each group, and the results are shown in Supplementary Figure S4.

To further test the hypothesis, the core body temperature over 24-h was monitored. WT mice exhibited a clear day/night oscillation and core body temperature peaked during the subjective night (active phase) and progressive decline to the lowest level during the light phase, whereas the body temperature of AD mice was markedly different losing the diurnal rhythm and fluctuated during the day. However, the oscillation pattern for GLP-1 sole, TRF sole, and combination groups was partially improved (Figure 2I).

### GLP-1 and TRF treatments improved the secretion rhythms of peripheral hormones of 5 × FAD mice

**FIGURE 3:**
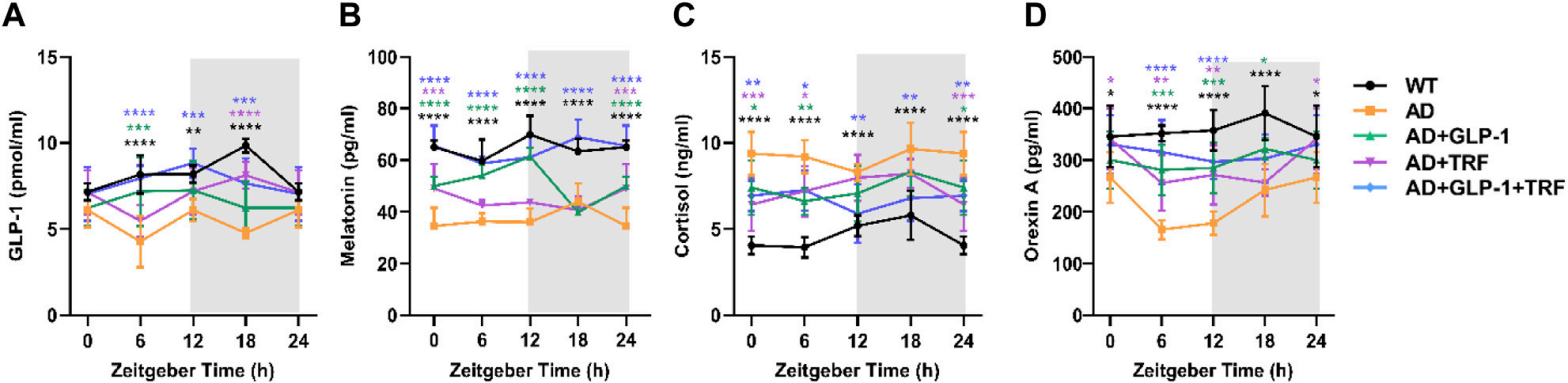
Influence of GLP-1and TRF on hormone secretion in mice. **(A–D)** Secretion levels of GLP-1, melatonin, cortisol, and orexin A in the five groups of mice detected by ELISA, respectively. *n* = 6 each group, *p < 0.05, **p < 0.01, ***p < 0.001, ****p < 0.0001 vs. AD group, using two-way ANOVA followed by Dunnett’s test. *n* = 6 per test and per group.

Circadian rhythms in concentrations of plasma GLP-1, cortisol, melatonin, and orexin A for five experimental groups are shown in Figure 3. Since animals for ZT 0 were sacrificed 24 h after Liraglutide injection, plasma GLP-1 was endogenous hormone secreted from the gut. Exogenous GLP-1 supplement (half-period:12 h) and TRF sole treatment induced endogenous secretion. The combination reached a better oscillation patter with a comparable level (Figure 3A). Consistent with previous study, secretion rhythm of melatonin for Black6 background mice was not apparent (Dauchy et al., 2019). As expected, the plasma melatonin level of AD mice was about half of that of WT mice. Surprisingly, combined treatment recovered the hormone level (Figure 3B).

Consistent with a previous study (Dauchy et al., 2019), the overall patterns of the daily plasma cortisol level rhythms of WT mice were low during the daytime phase and significantly higher during the dark phase, with the peak at ZT12 and decreasing to a nadir around ZT0. The cortisol rhythmicity was coincident with the daily activity. In contrast, oscillation pattern of plasma cortisol was disturbed with significant higher level in AD mice. GLP-1 sole, TFR sole, and combined treatments partially reduced the cortisol level at ZT12 (Figure 3C).

Orexin, a protein involved in regulating the sleep cycle and wakefulness. Significant reduction of orexin in CSF had been observed in patients with AD (Fronczek et al., 2012). In our study, plasma orexin A reached its peak during the subjective night, which corresponding to the high activity period. The level was elevated significantly via combined treatment of GLP-1 injection and TRF (Figure 3D).

### GLP-1 and TRF treatment improve the diurnal metabolic homeostasis of 5 × FAD mice

**FIGURE 4:**
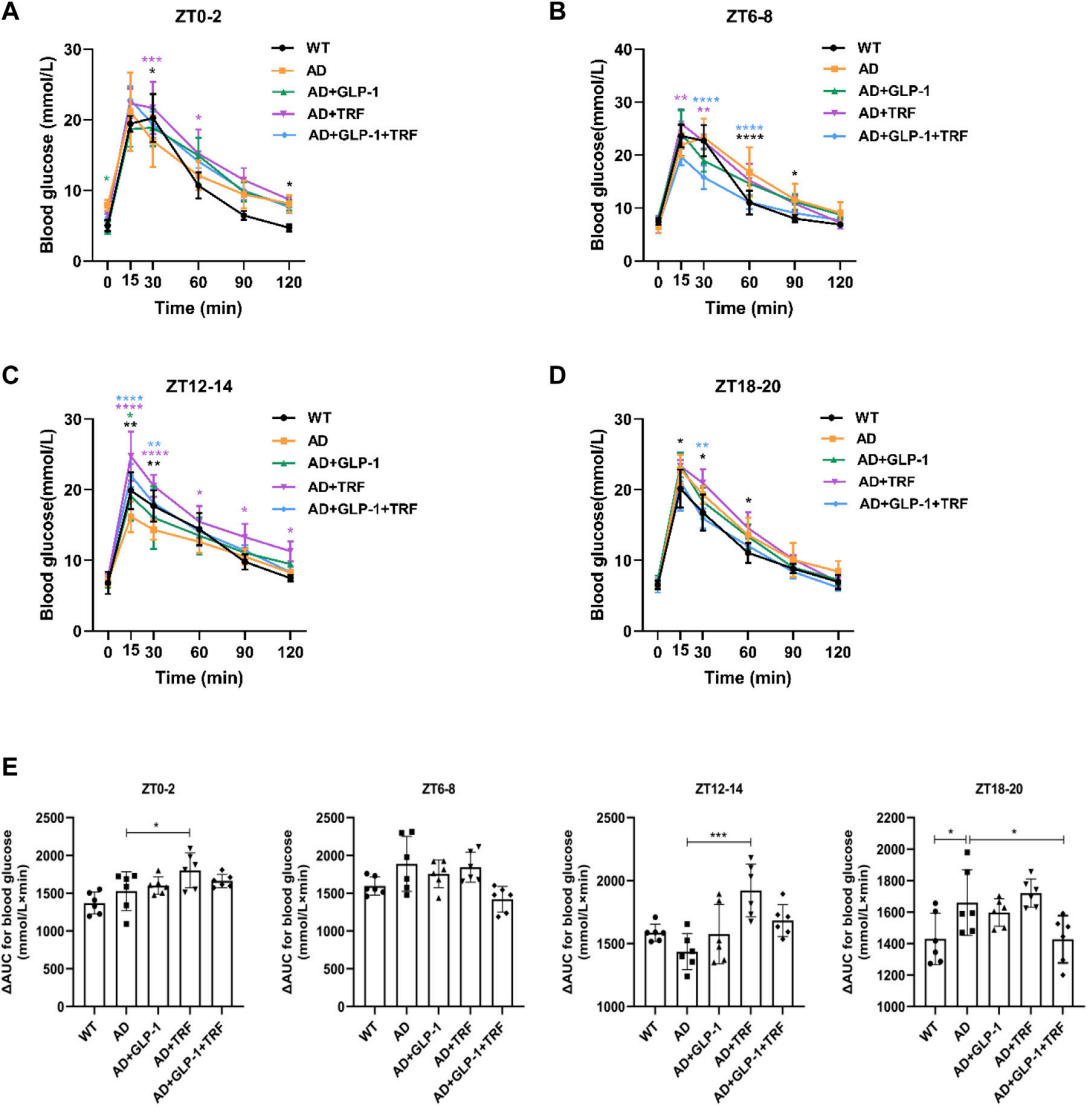
Effect of GLP-1and TRF on glucose metabolism. **(A–D)** Individual responses in blood glucose to identical intraperitoneal glucose tolerance test (IPGTT) conducted at four time points throughout the day (*n* = 6 per time point and per group). *p < 0.05, **p < 0.01, ***p < 0.001, ****p < 0.0001 compared to the AD group using two-way ANOVA followed by Dunnett’s test. **(E)** Corresponding ΔAUC for blood glucose. *n* = 6, *p < 0.05, ***p < 0.001 vs. AD group using One-Way ANOVA followed by Dunnett’s test.

At the end of the feeding protocol, intraperitoneal glucose tolerance tests (IPGTTs) were conducted with at a dose of 2 g glucose/kg after 16 h of fasting at ZT0, 6, 12, and 18. AD mice demonstrated significant glycemic abnormalities in response to an IPGTT (Figures 4A–D). At ZT12-14, the blood glucose of the AD group was lower than that of the WT group, and the opposite was true in the other three time periods, while that of TRF group was always at a higher level. At ZT12-14 and ZT18-20, dual treatment had a better effect on improving the glucose metabolism in AD mice. The area under the curve ΔAUC results showed that dual treatment helped to maintain the glucose homeostasis of ZT18-20 (ZT0-2: *p* = 0.0444, AD vs. AD + TRF. ZT12-14: *p* = 0.0001, AD vs. AD + TRF. ZT18-20: *p* = 0.0397, AD vs. WT; *p* = 0.037, AD vs. AD + TRF. Figure 4E).

### GLP-1 and TRF treatment improve spatial cognition and learning in 5 × FAD mice

**FIGURE 5:**
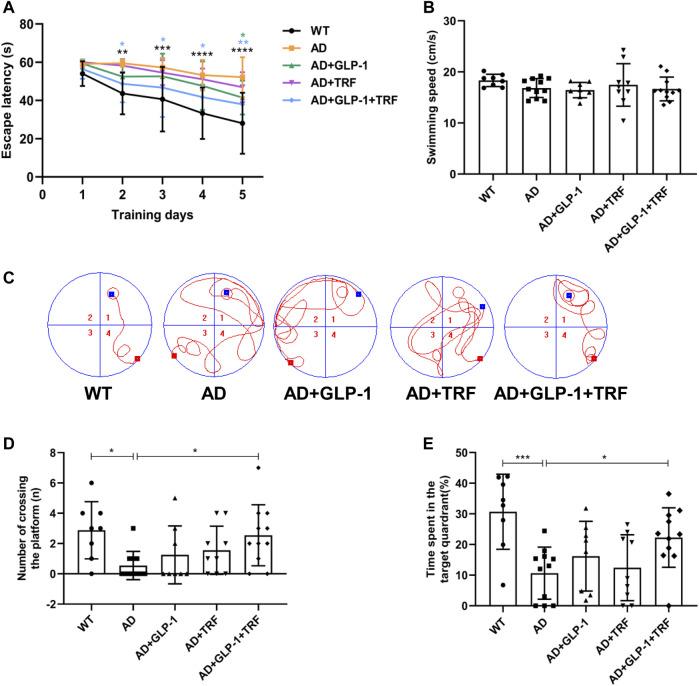
Changes in learning and memory capacity under the influence of GLP-1 and TRF. Cognition evaluation of the mice by the MWM. **(A)** Escape latency onto a hidden platform during the training trials of the Morris water maze test. *p < 0.05, **p < 0.01, ***p < 0.001, ****p < 0.0001 compared to the AD group, using One-Way ANOVA followed by Dunnett’s test or Dunn’s test. **(B)** Average swimming speed during the training trials. **(C)** Representative swimming tracks of the different groups of mice in the probe trial. **(D)** Numbers of crossing the platform during the probe trial. *p < 0.05 vs. AD group, using The Kruskal-Wallis test. **(E)** Percentage of time spent in the target quadrant in probe trial. *p < 0.05, ***p < 0.001 compared to the AD group, using one-way ANOVA followed by Dunnett’s test. *n* = 8–11 animals per group.

Cognitive impairment and memory deficits are typical clinical manifestations of Alzheimer’s disease (Chen et al., 2017; Fu et al., 2019). The Morris water maze (MWM) is generally considered to a validated test for the evaluation of the spatial learning and memory retention abilities of rats (Vorhees and Williams, 2006). A 6-day Morris water maze (MMW) test was performed according to a reported protocol (Vorhees and Williams, 2006) to evaluate the spatial learning and memory abilities of mice after 8 weeks of administration. In the probe trial, the AD mice exhibited impaired spatial cognition, as indicated by longer escape latencies. AD mice treated with both GLP-1 and TRF showed a significant shorter platform searching time. The mice in the dual treatment group showed a significant shorter platform searching time (Day 2: *p* = 0.0011, AD vs. WT; *p* = 0.0244, AD vs. AD + GLP-1 + TRF. Day 3: *p* = 0.0005, AD vs. WT; *p* = 0.0280. AD vs. AD + GLP-1 + TRF. Day 4: *p* < 0.0001, AD vs. WT; *p* = 0.0133, AD vs. AD + GLP-1 + TRF. Day 5: *p* < 0.0001, AD vs. WT; *p* = 0.0465, AD vs. AD + GLP-1; *p* = 0.0133, AD vs. AD + GLP-1 + TRF; Figure 5A), whereas mice in all groups showed no statistical difference in the mean swimming speed (Figure 5B). As expected, the AD mice traversed platforms less frequently and the mice treated with GLP-1 or/and TRF treatment made some progress, especially the dual treatment group (*p* = 0.0174, AD vs. WT; *p* = 0.0334, AD vs. AD + GLP-1 + TRF. Figure 5C). In probing trial, AD mice crossed the platform location less frequently, and WT and dual-treated mice crossed the platform more times (Figure 5D). Furthermore, the quadrant time analysis revealed that AD animals spent significantly shorter time in the target quadrant, whereas the dual treatment group animals increased time in the quadrant (*p* = 0.0006, AD vs. WT; *p* = 0.0423, AD vs. AD + GLP-1 + TRF. Figure 5E), indicating improved cognitive abilities. Unfortunately, the two groups of mice treated with GLP-1 and TRF monotherapy did not show significant changes in latency and number of plateaus crossed (Figures 5A,C,D). Therefore, in the follow-up experiments, we focused on the analysis of the AD mice with the dual treatment group.

### GLP-1 and TRF treatment improve attention and long-term memory in 5 × FAD mice

**FIGURE 6:**
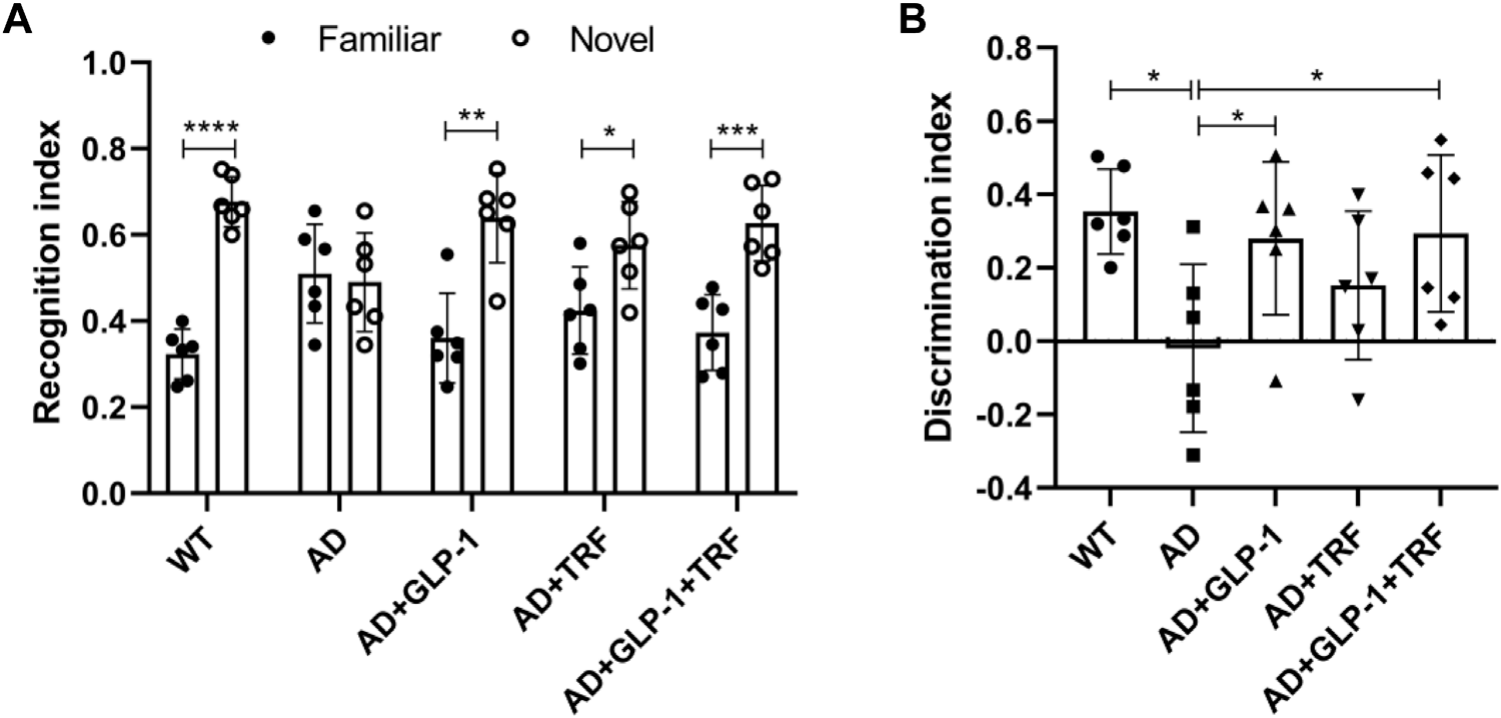
Novel object recognition task reveals improvement of GLP-1 and TRF on long-term memory and attention deficits in AD mice. **(A)** Recognition index was defined as the time to explore a familiar or new object/total time to explore both objects. *n* = 6, Two-tailed *t*-test, *p < 0.05, **p < 0.01, ***p < 0.001, ****p < 0.0001. **(B)** Discrimination index is calculated as the difference between the time spent exploring a new object and a familiar object/the total time spent exploring both objects. *n* = 6, One-Way ANOVA, *p < 0.05 vs. AD group.

We evaluate attention and long-term memory through the novel object recognition tasks. Except for the AD group, the other mice groups were able to discriminate between novel objects and familiar objects significantly (WT: *p* < 0.0001, AD: 0.7755, AD + GLP-1:0.0082, AD + TRF: 0.0262, AD + GLP-1+TRF: *p* < 0.0005, Two-tailed *t*-test, Figure 6A). Moreover, GLP-1 and combination treatment improved the discrimination index in AD mice, while TRF did not (F (4,25) = 3.381, *p* = 0.0242; *p* = 0.0112, AD vs. WT; *p* = 0.0491, AD vs. AD + GLP-1; *p* = 0.3882, AD vs. AD + TRF; *p* = 0.0378, AD vs. AD + GLP-1 + TRF. Figure 6B).

### GLP-1 and TRF combined treatment restored the tissue-specific kinetics of core clock gene transcripts of 5 × FAD mice

**FIGURE 7:**
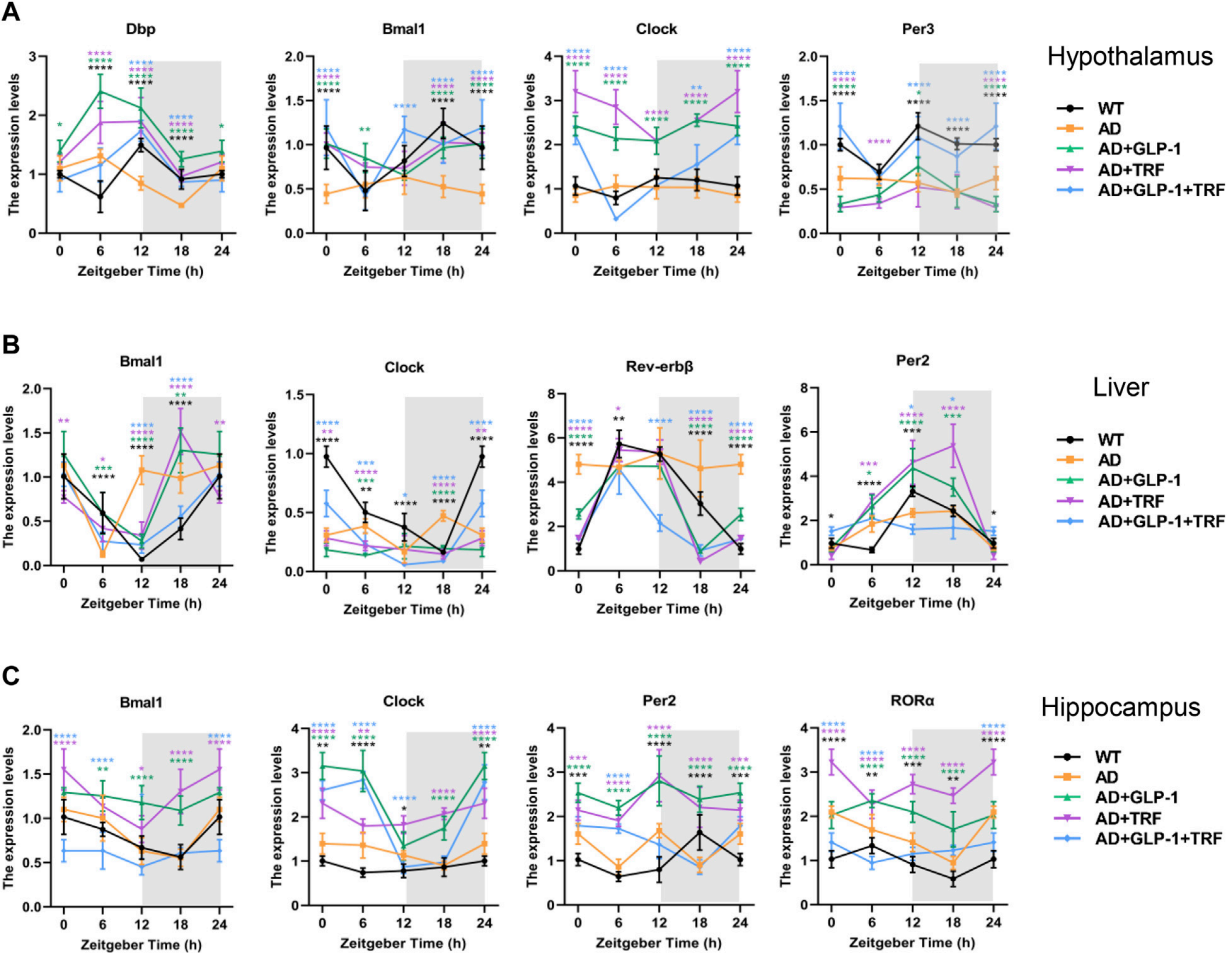
Impact of GLP-1 and TRF on circadian rhythm-related gene expression. **(A–C)** Relative expression level of circadian rhythm-related genes in the hypothalamus, liver and hippocampus tissues at four time points throughout the day, respectively. *p < 0.05, **p < 0.01, ***p < 0.001, ****p < 0.0001 vs. AD group, using two-way ANOVA followed by Dunnett’s test. n ≥ 5 per time point and per group.

To test whether cell-autonomous circadian clocks were entrained *via* dual treatment, we examined the mRNA expression profiles of the core clock genes. The hypothalamus was first detected, and the results were consistent with previous knowledge (Samaey et al., 2019). *Bmal1*, *Clock*, *Per3,* and *Dbp* rhythms were significantly disrupted in 5 × FAD mice. The three treatments altered the rhythm of core genes, and the rhythms of mRNA expression of *Dbp*, *Bmal1,* and *Per3* were significantly improved after double treatment (Figure 7A). In addition, mRNA expression rhythms of the core clock genes in the hippocampus and liver tissues were also examined. The results showed that the three treatments had better ameliorating effects on the circadian oscillation patterns of hepatic *Bmal1*, *Rev-erbβ,* and *Per2*. Dual treatment restored the rhythmic expression of *Clock*, while single treatment did not improve the gene (Figure 7B). For the hippocampus, the rhythm of *Bmal1* expression in 5 × FAD mice were consistent with that in normal mice, whereas the three treated groups were abnormal. The expression rhythm of *Clock*, *Per2,* and *RORa* in 5 × FAD mice was disturbed and changed to varying degrees after treatment, but did not show an expression pattern consistent with normal mice (Figure 7C).

### GLP-1 and TRF combined treatment ameliorated the neurodegeneration pathogenesis of 5 × FAD mice

**FIGURE 8:**
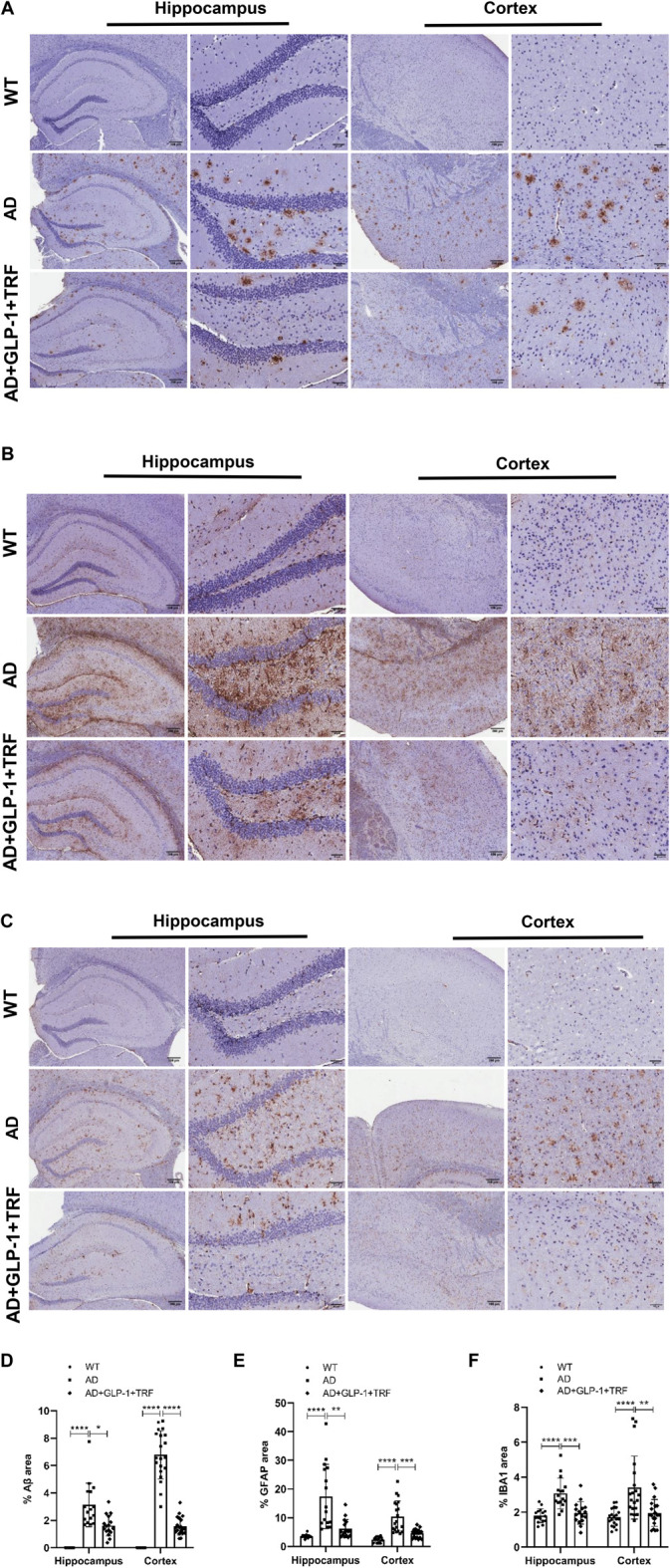
Role of GLP-1 and TRF on induced decrease in Aβ, GFAP, and IBA1 expression. **(A–C)** Representative hippocampus and cortex images from three groups stained for Aβ (MOAB-2), GFAP, and (IBA1), including a 4× field and a 20× field. Scale bars are 200 and 50 μm respectively. **(D–F)** The quantitative analysis of the positive areas of the three proteins in the hippocampal and cortical regions, respectively. Hippocampus: Averages of 4 fields per slice, *n* = 4 animals per group were quantified. Cortex: Averages of 5 fields per slice were quantified. *n* = 4. *p < 0.05, **p < 0.01, ***p < 0.001, ****p < 0.0001. Aβ: The Kruskal-Wallis test. GFAP: The Kruskal-Wallis test was used for quantitative analysis of hippocampal regions and Brown-Forsythe and Welch ANOVA tests for cortical regions. IBA1 of hippocampus: One-Way ANOVA followed by Dunnett’s test. IBA1 of cortex: The Kruskal-Wallis test.

In addition to pathological hallmarks, AD is accompanied by prominent neuroinflammation, manifested by microglia hyperplasia, reactive astrocyte hyperplasia (Litvinchuk et al., 2018; Lananna et al., 2020). Both the hippocampus and cortex tissue sections were examined (Figures 8A–C). We observed that GLP-1 administration and TRF combination ameliorated the Aβ deposits significantly in both the hippocampus and cortex tissue (*Hippocampus*: *p* < 0.0001, AD vs. WT; *p* = 0.0384, AD vs. AD + GLP-1 + TRF. Cortex: *p* < 0.0001, AD vs. WT and AD vs. AD + GLP-1 + TRF; Figure 8D), suggesting the possible mechanism for improved spatial cognition and learning of 5 × FAD mice. Furthermore, chronic glia activation played an important role during AD pathogenesis and contributed to neurotoxicity and synapse loss. Glial fibrillary acidic protein (GFAP), a marker of astrocyte activation, and ionized calcium-binding adapter molecule 1 (IBA1), a marker of microglia, were examined for the effect of combined treatment on glial activation. Remarkable increased staining for GFAP was observed in both the hippocampus and cortex regions of 5 × FAD mice (*Hippocampus*: *p* < 0.0001, AD vs. WT; *p* = 0.0049, AD vs. AD + GLP-1 + TRF. Cortex: *p* < 0.0001, AD vs. WT; *p* = 0.0003, AD vs. AD + GLP1 + TRF; Figure 8E). Combined treatment reduced GPAP, however, the reduction of GFAP signal of cortex region was more prominent than that of the hippocampus. Moreover, decreased staining for IBA1 in both the hippocampus and cortex regions was observed (*Hippocampus*: *p* < 0.0001, AD vs. WT; *p* = 0.0006, AD vs. AD + GLP-1 + TRF. Cortex: *p* < 0.0002, AD vs. WT; *p* < 0.0017, AD vs. AD + GLP-1 + TRF; Figure 8F). Immunohistochemical analysis showed that long-term GLP-1 application and TRF inhibited astrocyte and microglial activation in the brain of 5 × FAD mice.

## Discussion

More than half of AD patients disturbed circadian rhythm and metabolic problem. An altered sleep–wake pattern negatively impacts amyloid burden and exacerbated neurotoxicity and decreases the self-care ability of patients. Circadian disturbance and AD may be a reciprocal causation: neurodegeneration likely impairs circadian rhythms either by dysfunctional SCN neuron or abnormal communication between the central clock with the peripheral tissues (Chauhan et al., 2017; Van Erum et al., 2018). Moreover, hypoglycemia is observed with AD patients in early stage (Cunnane et al., 2011) and diabetes and impaired insulin signaling further worsen the pathogenesis of AD. Circadian arrhythmia and metabolic defects together increase the sensitivity of the brain to further degeneration processes of AD (Musiek and Holtzman, 2016; Oh et al., 2019). In our study, we observed serious arrhythmia of 5 × FAD mice, including the altered activity–rest cycle, peripheral Per2 expression, and GLP-1 secretion. We had confirmed that GLP-1 administration together with time-restricted feeding improve the circadian rhythm of 5 × FAD mice including the rhythm of activity–rest cycle, feeding–fasting cycle, and core body temperature. On the one hand, 5×FAD mice had reduced daytime activity and meal duration after 2 months of treatment. On the other hand, cosinor analysis showed that AD mice had lower amplitudes and later peaks in activity, and increased amplitudes and earlier peaks after single and dual treatments. For the feeding cycles, GLP-1 reduced mesor in AD mice, but there were no significant between-group differences in amplitude and phase. Furthermore, GLP-1 and TRF treatments improved the diurnal metabolic homeostasis and hormone secretion of 5 × FAD mice. MWM and NOR tests showed that GLP-1 combined with TRF can significantly improve the spatial learning and cognitive ability and long-term memory ability of AD mice, while the single treatment has no good effect. The abnormal expression of clock genes, including *Baml1*, *Clock,* and *Dbp,* were improved in the hypothalamus, and ameliorated pathogenesis of neurodegeneration was also observed in AD mice with dual treatment. It is worth noting that the daily food intake of the mice in the TRF group and the dual treatment group was lower than that in the other three groups, which suggest that TRF reduced the caloric intake of the mice. Thus, we cannot rule out that total food intake has an effect on the circadian rhythm and metabolic effects. But the time-restricted feeding regimen did improve the activity rhythms, hormone secretion, and cognitive function in AD mice. Further experiments are needed to study the relationship among duration of TRF, food consumption, and circadian rhythm.

Circadian control relies on the coordination between the central clock and peripheral clock. The SCN neurons are synchronized mainly *via* light cues transmitted from the retina, and *via* neuronal and humoral cues SCN transduce daytime information to peripheral organs. Furthermore, feeding–fasting cycles appear to be the dominant Zeitgeber for peripheral organs, such as liver, adipose tissue, skeleton muscle, and heart (Dibner et al., 2010; Sato et al., 2017; Reinke and Asher, 2019). Along with the disrupted activity–rest cycle, 5 × FAD mice exhibit altered feeding–fasting cycle with increased feeding time during the light phase. Therefore, we hypothesized that long-term resetting feeding–fasting cycle might affect the circadian regulation system of AD mice. Interestingly, we observed that 60 days long term dual treatment had improved the expression rhythms of the core clocks in the hypothalamus.

After detecting the secretion level of GLP-1, we explored the glucose metabolism status of 5 × FAD mice by the IPGTT experiment. The results showed that in the ZT0-2 experiment, the blood sugar level of AD mice was higher at 30 min, and the blood sugar level in the treatment group was decreased, especially in the dual treatment group. However, the results of the IPGTT experiment in the four time periods showed that there was no statistical difference in the area under the blood glucose curve of the different groups of mice, which indicated that the AD mice used in this study did not exhibit abnormal glucose tolerance. More research is needed to explore the occurrence time and specific manifestations of abnormal glucose tolerance in 5 × FAD mice.

Given that hypoglycemia is observed in the brain of AD patients in early stage, AD is also called intracerebral diabetes (Cunnane et al., 2011). Diabetes and impaired insulin signaling in the brain are linked to the pathogenesis of AD (Chauhan et al., 2017). The changes in sleep and eating rhythms make the central and peripheral metabolisms out of synchrony, causing a decrease in brain protein clearance, which may lead to the prevalence of metabolic syndrome and/or diabetes (Chauhan et al., 2017). GLP-1, a hormone secreted from the gut, can promote insulin secretion, delay gastric emptying, and inhibit appetite. The plasma level of GLP-1 was significantly decreased, and the secretion rhythm was disrupted in 5 × FAD mice. In order to suppress abnormal feeding during the light phase, the exogenous GLP-1 injection was administrated at ZT0. Surprisingly, exogenous GLP-1 sole treatment did improve the activity–rest and feeding–fasting cycle significantly. Daily rhythm of core body temperature was partially recovered. Interestingly, daily rhythm of endogenous GLP-1 secretion was improving not only by exogenous application but also by TRF.

Time-restricted feeding, as an emerging dietary intervention, restricts food intake to a certain time period of the day and forces to make the daily eating–fasting cycle consistent (Wilkinson et al., 2020). TRF drives changes in behavioral rhythms, such as increased body temperature and metabolic disorders (Bray et al., 2013; McHill et al., 2017). Furthermore, limiting eating to certain phase has been proven to reset the clocks in the various peripheral organs and brain areas, although not in the SCN (Damiola et al., 2000; Feillet et al., 2008). In our study, TRF improved the activity–rest rhythms, body temperature rhythm, and oscillated hormone secretion rhythm such as GLP-1 and cortisol. Dual treatment significantly improved learning and cognitive abilities in AD mice, while the two groups treated with monotherapy did not. It may take longer treatment time to get the desired effect. Surprisingly, long-term TRF combined with exogenous GLP-1 injection ameliorated the rhythm of the core clock gene expression in the hypothalamus and liver, but not in the hippocampus, suggesting that the tissues of the central nervous system are less easily entrained or destroyed by various feeding patterns. Since the results of the water maze experiment showed that dual administration significantly improved the learning and cognitive abilities of the 5 × FAD mice, and at the same time restored the rhythmic oscillation of the mRNA expression of some core clock genes in the hypothalamus and liver, therefore, in the follow-up experiments, we focused on the AD mice in the double treatment group. Combination treatment reduced Aβ plaques and glial cell activation in the AD mouse brain tissue significantly, which was consistent with the results of the water maze experiment. Sleep is essential for maintaining the CSF flow coupled with hemodynamic oscillations, which is important for protecting the healthy brain (Fultz et al., 2019). Along with the reduction of overall activity during the subjective day time and less fragmented rest period, GLP-1 supplementation combined with TRF might activate protective mechanism to clean the amyloid deposits. However, further experiments were required to uncover the underlined mechanism.

In summary, this study demonstrated for the first time that circadian rhythm disorders of AD mice could be improved *via* feeding restriction together with GLP-1 injection. Long-term treatment even altered the rhythmic pattern of core clock genes in the hypothalamus. It further clarified that the altered feeding–fasting pattern of AD mice could alleviate metabolic defects and the neurodegeneration progression *via* resetting the circadian clock. Our study provides a novel possible treatment of AD patients with circadian rhythm disorders.

## Data Availability

The original contributions presented in the study are included in the article/Supplementary Material. Further inquiries can be directed to the corresponding author.
