# Corrigendum: Resetting the circadian clock of Alzheimer’s mice *via* GLP-1 injection combined with time-restricted feeding

**DOI:** 10.3389/fphys.2022.1077956

**Published:** 2022-11-21

**Authors:** Yanqiong Dong, Le Cheng, Yingying Zhao

**Affiliations:** ^1^ Department of Basic Medicine Sciences, School of Basic Medical Sciences, Dali University, Dali, Yunnan, China; ^2^ Department of Physiology, School of Basic Medical Sciences, Shenzhen University Health Sciences Center, Shenzhen, Guangdong, China; ^3^ BGI-Yunnan, BGI-Shenzhen, Kunming, Yunnan, China

**Keywords:** amyloid-β, circadian rhythm, glucagon-like peptide-1, time-restricted feeding, Alzheimer’s disease

Error in Figure/Table

In the published article, there was an error in Figure 1B as published. Figure 1B is not fully displayed. The corrected Figure 1B and its caption **Alzheimer’s disease mice exhibit circadian rhythm disturbances** appear below.

**FIGURE 1:**
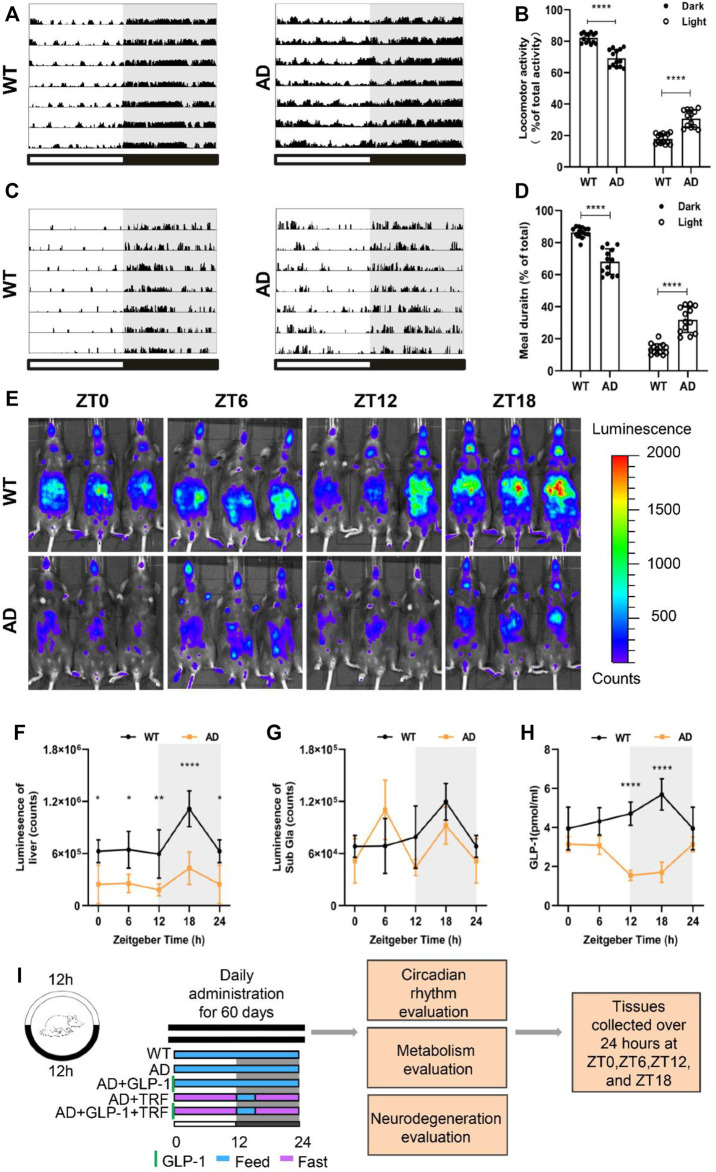
Alzheimer’s disease mice exhibit circadian rhythm disturbances. **(A)** Representative locomotor activity records of 5 × FAD mice (AD) and wildtype mice (WT), respectively. Locomotor activity was defined as the moving distance per unit time (2 min). Each horizontal line represents 24 h. Periods of darkness are indicated by grey backgrounds. The black and white bars on the button indicate 12 h-dark and 12 h-light periods, respectively. **(B)** Percentage of the locomotor activity in the dark and light/total activity (24 h). *n* ⁼ 7 per group, ****p 0.0001, using two-way ANOVA followed by Sidak t test. **(C)** Representative meal duration records of two groups. **(D)** Percentage of the meal duration in the dark and light/total meal duration (24 h). *n* ⁼ 7 per group, ****p 0.0001, using Two-Way ANOVA followed by Sidak t test. **(E)** Representative photographs of PER2:LUC mice in vivo monitoring from each time point at 6 h intervals. **(F,G)** Raw photon count data of individual bioluminescence rhythms from E. *n* ⁼ 3 per group, *n* ⁼ 3 per group, *p 0.015, **p < 0.01 using Two-Way ANOVA test. **(H)** GLP-1 secretion levels in both groups. **(I)** The protocol design of our study.

The authors apologize for this error and state that this does not change the scientific conclusions of the article in any way. The original article has been updated.

