# Supplementary material for: Resetting the circadian clock of Alzheimer’s mice via GLP-1 injection combined with time-restricted feeding
Source: Front Physiol. 2022 Aug 24;13:911437. doi: 10.3389/fphys.2022.911437 (PMC9487156; doi:10.3389/fphys.2022.911437)
Supplement: Supplementary file 1 [file Presentation1.pdf]

Supplementary Figure Legends

Fig. S1 The impact of saline and TRF on circadian rhythm. (A) Representative locomotor activity records of each group. Representative locomotor activity records of intraperitoneal injection of saline (AD + Sal) and TRF combined with intraperitoneal injection of saline (AD + TRF + Sal) in AD mice. (B) Locomotor activity during the light phase (m). n=7 per group, *** $p < 0.001$ vs AD group using One-Way ANOVA followed by Dunnett's test. (C) Activity during the dark phase (m). n=7 per group. (D) The ratio of the activity in the dark and light/total activity in each group. n=7 per group. *** $p < 0.001$ vs AD mice using Two-Way ANOVA followed by Dunnett's test. (E) Representative meal duration records of each group. (F) Meal duration during the dark phase (s). n=7 per group.

Fig. S2 Cosinor analysis of activity rhythms in mice. (A-E) are the periodogram analysis of WT, AD, AD+GLP-1, AD+TRF and AD+GLP-1+TRF, respectively. n = 5 each group. (F-I) are the mesor, amplitudes, acrophase and bathyphase of all groups, respectively. n = 5 per group, One-Way ANOVA followed by Dunnett's test. * $p < 0.05$, *** $p < 0.001$, **** $p < 0.0001$.

Fig. S3 Cosinor analysis of feeding rhythms in mice. (A-C) are the periodogram analysis of WT, AD and AD+GLP-1, respectively. n = 5 each group. (D-F) are the mesor, amplitudes, acrophase and bathyphase of the three groups, respectively. n = 5. One-Way ANOVA followed by Dunnett's test. ** $p < 0.01$, *** $p < 0.001$.

Fig. S4 The daily food intake of mice in all groups. n = 21. One-Way ANOVA followed by Dunnett's test. *** $p < 0.001$, **** $p < 0.0001$.
