# Supplementary figures and images for: Resetting the circadian clock of Alzheimer’s mice via GLP-1 injection combined with time-restricted feeding
Source: Front Physiol. 2022 Aug 24;13:911437. doi: 10.3389/fphys.2022.911437 (PMC9487156; doi:10.3389/fphys.2022.911437)

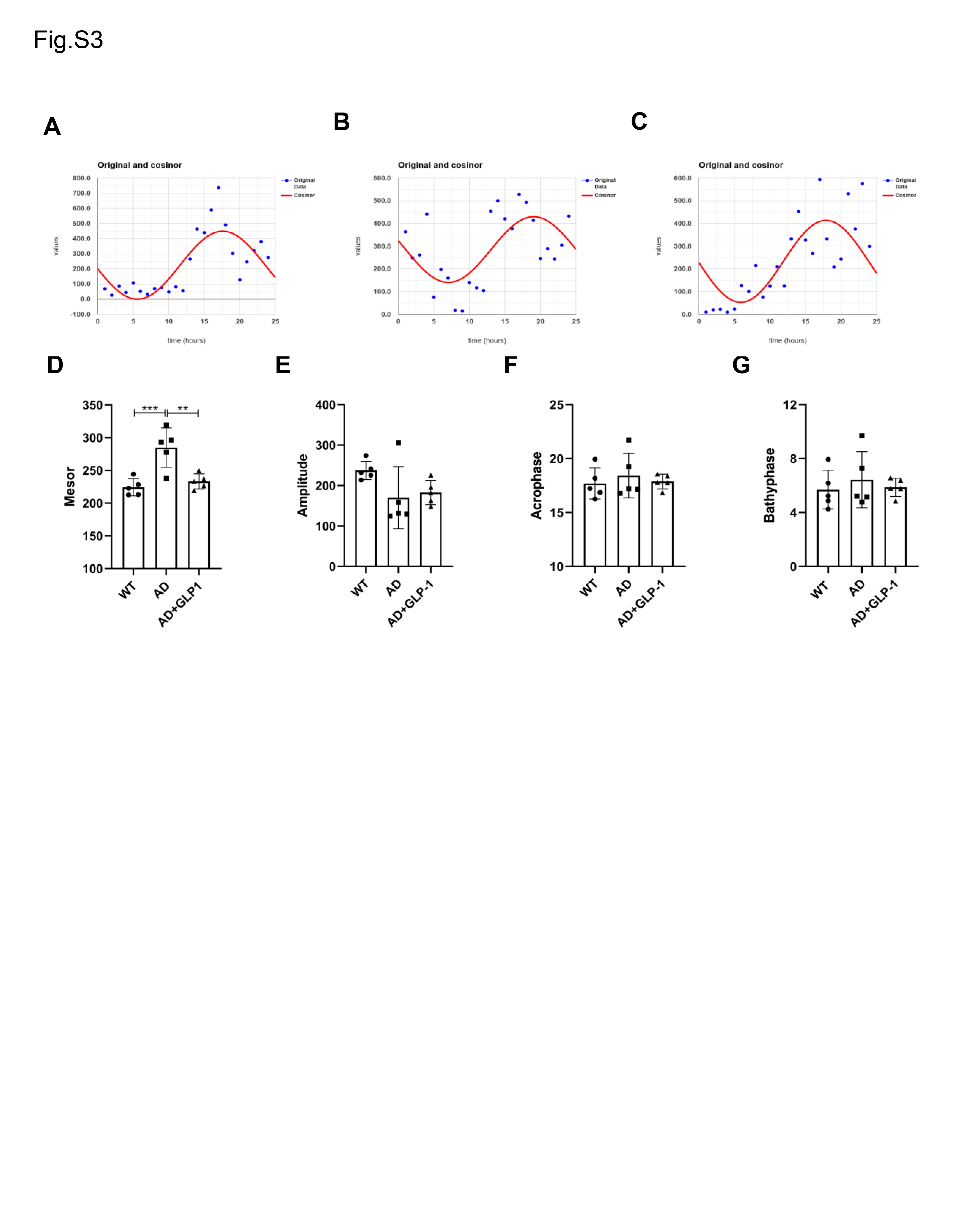

Supplement: Supplementary file 2 [file Image3.tif]

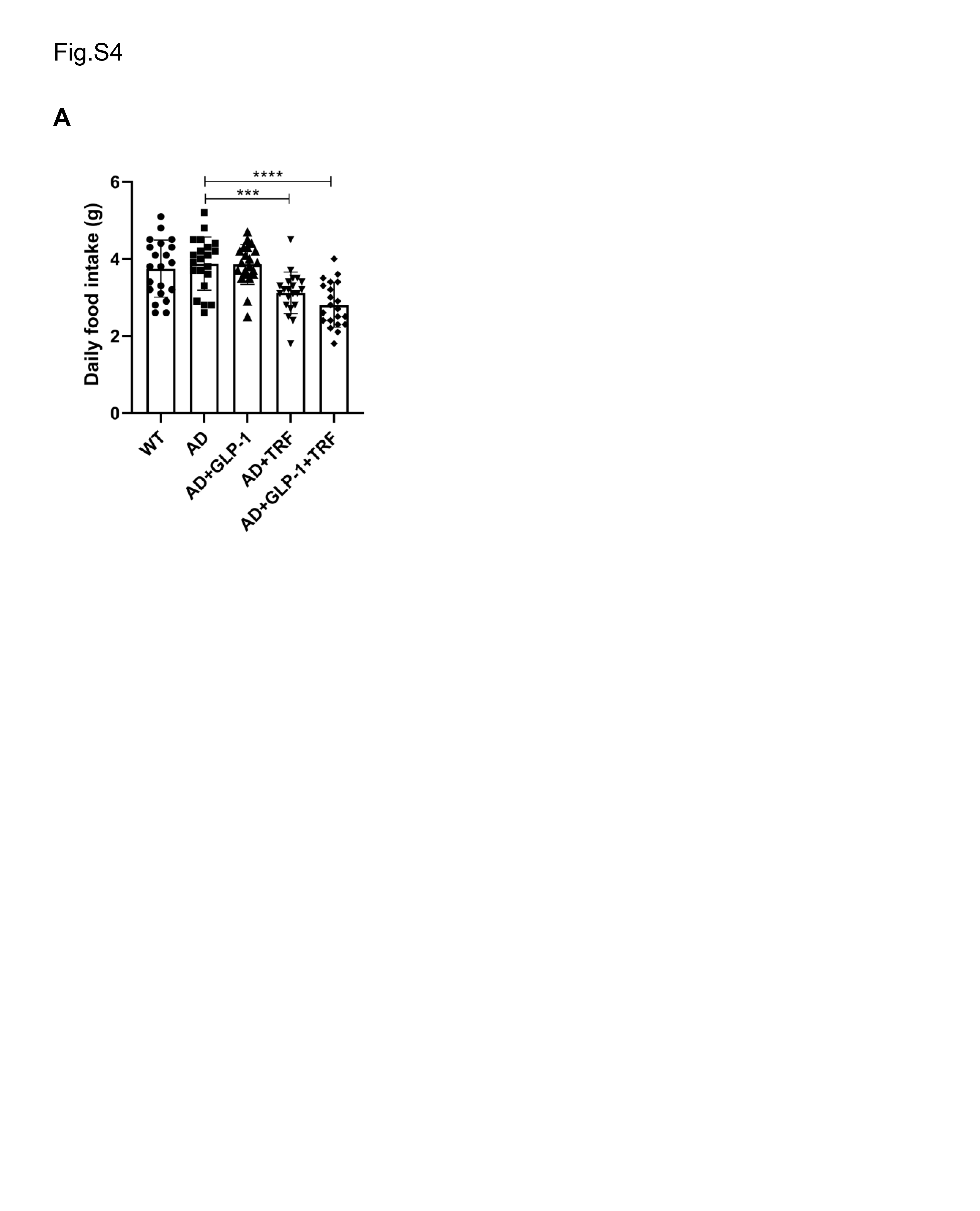

Supplement: Supplementary file 3 [file Image4.tif]

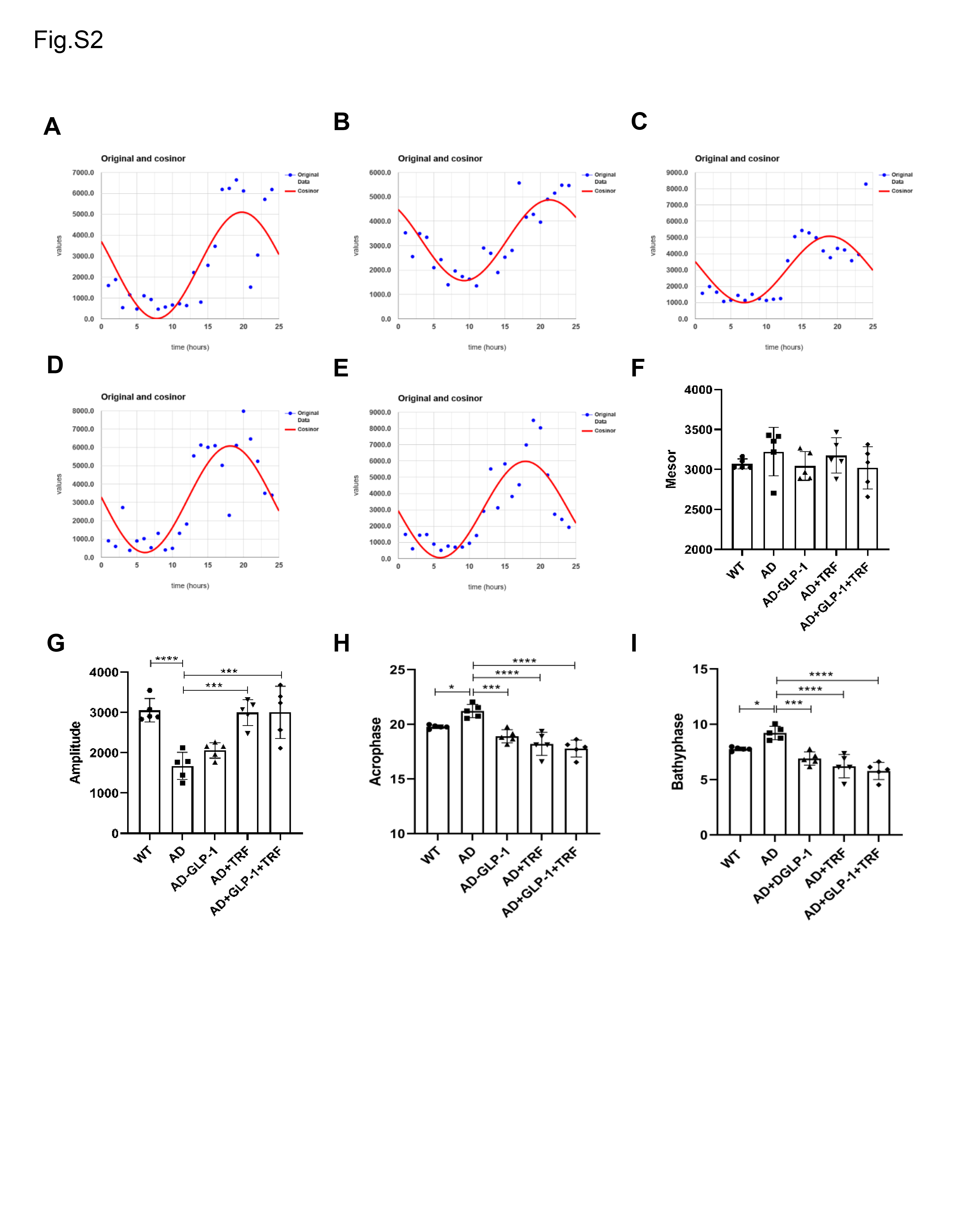

Supplement: Supplementary file 4 [file Image2.tif]

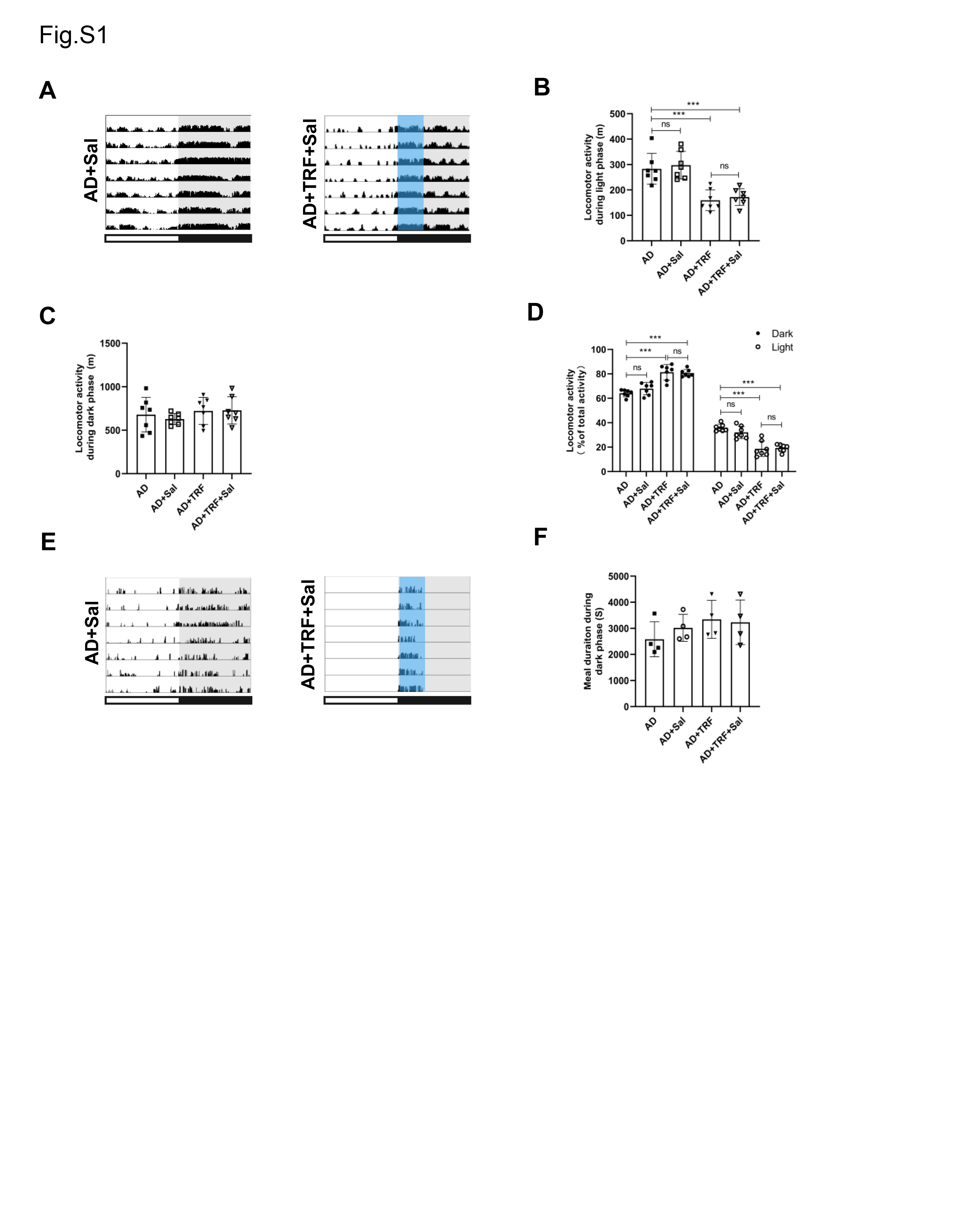

Supplement: Supplementary file 5 [file Image1.tif]
